# Punicalagin Protects against the Development of Methotrexate-Induced Hepatotoxicity in Mice via Activating Nrf2 Signaling and Decreasing Oxidative Stress, Inflammation, and Cell Death

**DOI:** 10.3390/ijms232012334

**Published:** 2022-10-15

**Authors:** Alayn’ Al-marddyah A. Al-khawalde, Mohammad H. Abukhalil, Muthana M. Jghef, Manal A. Alfwuaires, Fatima S. Alaryani, Saleem H. Aladaileh, Abdulmohsen I. Algefare, Shaik Karimulla, Fawaz Alasmari, Hammad Khalifeh Aldal’in, Abdulkareem A. Alanezi, Osama Y. Althunibat

**Affiliations:** 1 Department of Medical Analysis, Princess Aisha Bint Al-Hussein College of Nursing and Health Sciences, Al-Hussein Bin Talal University, Ma’an 71111, Jordan; 2 Department of Biology, College of Science, Al-Hussein Bin Talal University, Ma’an 71111, Jordan; 3 Department of Radiology, College of Medical Technology, Al-Kitab University, Kirkuk 36001, Iraq; 4 Department of Biological Sciences, Faculty of Science, King Faisal University, Al-Ahsa 31982, Saudi Arabia; 5 Department of Biology, College of Science, University of Jeddah, Jeddah 21589, Saudi Arabia; 6 Department of Pharmacy Practice, College of Pharmacy, University of Hafr Al-Batin, Hafr Al-Batin 31991, Saudi Arabia; 7 Department of Pharmacology and Toxicology, College of Pharmacy, King Saud University, Riyadh 11451, Saudi Arabia; 8 Department of Medical Support, Al-Karak University College, Al-Balqa Applied University, Al-Karak 19117, Jordan; 9 Department of Pharmaceutics, College of Pharmacy, University of Hafr Al-Batin, Hafr Al-Batin 31991, Saudi Arabia

**Keywords:** methotrexate, oxidative stress, Nrf2, hepatotoxicity, punicalagin

## Abstract

Despite its effectiveness in treating inflammatory diseases and various malignancies, methotrexate (MTX) is well known to cause hepatotoxicity, which involves increased oxidative stress and inflammation, limiting its clinical use. Herein, we looked into the effect of punicalagin (PU), a polyphenolic molecule having a variety of health-promoting attributes, on MTX-induced hepatotoxicity in mice. PU (25 and 50 mg/kg/day) was given orally to the mice for 10 days, while a single dose of MTX (20 mg/kg) was injected intraperitoneally (i.p.) at day 7. The MTX-induced liver damage was demonstrated by remarkably higher transaminases (ALT and AST), ALP, and LDH, as well as significant histological alterations in hepatic tissues. MTX-injected mice also demonstrated increases in hepatic oxidative stress markers, including malondialdehyde (MDA) and nitric oxide (NO), with a concordant drop in glutathione (GSH) content and superoxide dismutase (SOD) and catalase (CAT) activities. PU significantly attenuated the MTX-induced serum transaminases, ALP and LDH elevations, and hepatic oxidative stress measures and boosted antioxidant defenses in the liver. Moreover, the liver of MTX-treated mice showed increases in NF-κB p65 expression, pro-inflammatory cytokine (IL-6 and TNF-α) levels, and pro-apoptotic protein (caspase-3 and Bax) expression, whereas Bcl-2 and Nrf2 expressions were reduced, which were all attenuated by PU treatment. Collectively, PU inhibits oxidative damage, inflammation, and apoptosis and upregulates Nrf2 in the liver of MTX-induced mice. Thus, these findings suggest that PU may have great therapeutic potential for the prevention of MTX-induced hepatotoxicity, pending further exploration in upcoming studies.

## 1. Introduction

Drug-induced liver injury (DILI) is a term used to describe any injury to the liver by drugs or other xenobiotics that can lead to chronic liver disease and/or acute liver failure, with an estimated incidence between 13.9–19.1 cases per 100,000 persons exposed per year [1,2]. The two mechanisms of DILI are intrinsic, which is dose-dependent and occurs shortly after exposure, and idiosyncratic, which is more unpredictable and has a longer latency period [2]. Liver toxicity related to drugs can mimic any form of acute or chronic hepatobiliary conditions, such as acute viral hepatitis, biliary obstruction, acute fatty liver and chronic hepatitis, and cirrhosis [3]. A plethora of drugs can cause liver injury, including anticancer drugs, anesthetics, antimicrobial medications, non-steroidal anti-inflammatory drugs, and acetaminophen, among others [2,3]. Among hepatotoxic drugs, methotrexate (MTX) is a commonly used anticancer and immunosuppressive drug for treating a variety of malignancies and autoimmune diseases [4,5,6,7]. It is a folate antimetabolite that can cause significant toxicity, including hepatotoxicity, acute kidney injury (AKI), pulmonary damage, myelosuppression, and mucositis, limiting its clinical usage [8,9,10]. Although chronic MTX toxicity is more documented in patients receiving MTX, acute MTX toxicity can be a life-threatening emergency in the form of multiorgan failure [11,12]. Hence, there is a need to develop effective pharmacotherapies to prevent and decrease the devastating complications of MTX, including hepatotoxicity.

Following its administration, MTX distributes to extravascular compartments, including synovial fluid, and to the non-fatty tissues of the body, including liver, kidney, and joint tissues [13,14]. In some organs (e.g., the liver and kidney), MTX can be converted by folylpolyglutamate synthetase to active polyglutamate derivatives which are selectively retained in cells longer than MTX [13,15]. Moreover, MTX partly undergoes hydroxylation by hepatic aldehyde oxidase to 7-hydroxymethotrexate with a long half-life of 24 h in humans [16,17]. MTX is mainly excreted by the kidney as a result of both glomerular filtration and tubular secretion. Extrarenal routes of MTX excretion, including biliary excretion and secretion into human breast milk and saliva, may occur [13,18]. Even though the molecular mechanisms behind the hepatotoxicity of MTX have not yet been fully understood, experimental and clinical investigations are consistent with the idea that excessive reactive oxygen species (ROS) generation, oxidative stress, inflammation, DNA damage, and caspase-3 activation play a key role in the development of MTX hepatotoxicity [8,9,15,19]. It has been reported that MTX metabolites, including MTX-polyglutamate, can cause ROS overproduction, steatosis, and fibrosis in the liver [15]. Indeed, increased ROS production induces oxidative damage to DNA, lipids, and proteins, as well as an inflammatory response via nuclear factor kappa-B (NF-κB) activation and the production of pro-inflammatory cytokines, resulting in liver apoptosis and injury [10,15,20,21,22]. Thus, the activation of antioxidant and cytoprotective pathways can show a robust protective strategy against the development of MTX hepatotoxicity. Among these protective strategies is the activation of the redox-regulated transcription factor nuclear factor (erythroid-derived 2)-like 2 (Nrf2), which can protect against cellular oxidative damage through the regulation of the basal and inducible expression of a plethora of antioxidant and cytoprotective genes [23,24]. Therefore, the activation of Nrf2 may show a promising feasible approach for the prevention/treatment of MTX-induced liver injury.

Extensive evidence suggests that using natural substances with antioxidant and anti-inflammatory characteristics can effectively treat MTX-induced liver injuries [25,26,27,28]. Plants are considered as one of the main sources of medically relevant active compounds, including polyphenols. Among them, the pomegranate (*Punica granatum* L.) is a small tree that is cultivated in the tropics and deciduous in subtropical and temperate zone areas [29]. It possesses high amounts of phenolic compounds, including hydrolyzable tannins, flavonol glucosides, phenolic acids, ellagic acid derivatives, and flavonoids in its various parts such as fruits, seeds, leaves, and peels [30,31]. Pomegranate is known for its potential health-promoting properties, including antioxidant, anti-inflammatory, anticancer, antihypertensive, antiatherosclerosis and antimicrobial activities [29,32]. Punicalagin (PU, C_48_H_28_O_30_) is one of the major active pomegranate polyphenols possessing antioxidant and anti-inflammatory properties, among others [33,34,35]. PU was shown to attenuate diabetes-induced cardiac pathology in rats via modulating myocardial oxidative injury, inflammation, and apoptosis [36]. It has also been demonstrated that PU attenuated acetaminophen-induced liver damage and histological changes by reducing oxidative stress [37]. PU was reported to protect against diabetic liver injury in mice through the attenuation of oxidative stress and restoration of antioxidants [38]. Moreover, PU prevented cyclophosphamide-induced liver injury by inhibiting oxidative/nitrosative stress, inflammation, and apoptosis [39]. PU also showed a protective action against acrylamide-induced neurotoxicity and hepatotoxicity by modulating oxidative stress and apoptosis in rats [40]. PU protected rats against cisplatin-induced renal damage by lowering oxidative stress, inflammation, and apoptosis while increasing Nrf2 and antioxidant levels [34]. Moreover, another study found that PU mitigated carbon tetrachloride (CCl_4_)-induced liver damage by improving antioxidative activities and autophagy via the Akt/FOXO3a and P62/Nrf2-signaling pathways [41]. In addition, PU attenuated methionine-induced brain damage [42] and protected against streptozotocin-induced pancreatic injury and insulitis [43] in rodents by modulating oxidative stress, inflammation and apoptotic cell death. PU, like all other ellagitannins, is hydrolyzed in the small intestine to form ellagic acid, which is then metabolized by intestinal bacteria via numerous decarboxylation steps to produce urolithins [44]. Similar to PU, ellagic acid and urolithins have shown significant antioxidant activity and protective effects against oxidative stress-mediated tissue damage [45,46,47,48]. While PU and ellagic acid are poorly bioavailable due to their rapid degradation or low water solubility, urolithins are the most bioavailable ellagitannin derivatives absorbed from the intestine and distributed in the body fluids and tissues, appearing as the actual metabolites responsible for beneficial bioactivities obtained from PU and other ellagitannins ingestion [49,50].

Despite its multiple therapeutic effects, the protective action of PU against MTX-induced hepatotoxicity is yet to be studied. Therefore, we hypothesized that treatment with PU would be a novel strategy for protecting the liver from the adverse effects of MTX. In this study, we have investigated the effect of PU on oxidative stress, inflammation, and apoptosis, hinting at a putative role for Nrf2 in the protection of MTX-induced hepatotoxicity in mice. This study underscores the potential of PU for the prevention of hepatotoxicity induced by MTX.

## 2. Results

### 2.1. PU Prevents MTX-Induced Liver Injury in Mice

**Figure 1:**
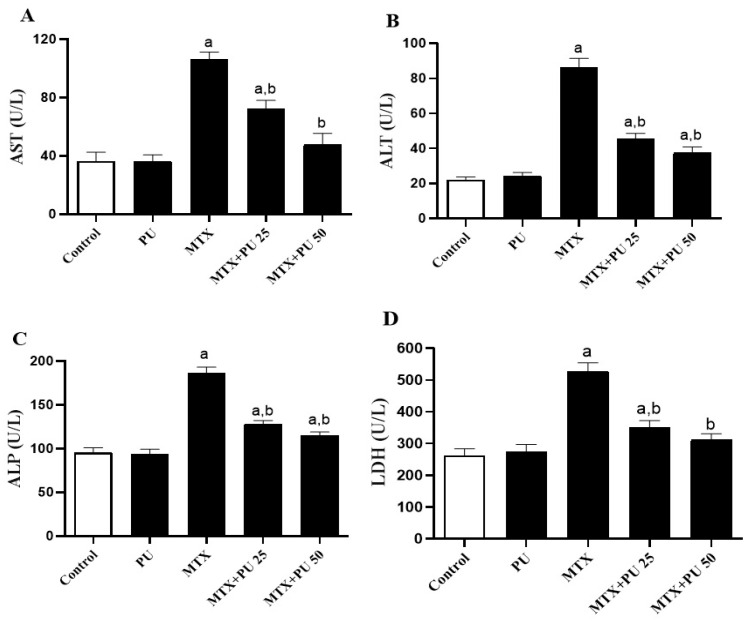
PU ameliorates liver function in mice exposed to MTX. PU reduced the blood levels of (**A**) AST, (**B**) ALT, (**C**) ALP, and (**D**) LDH activities in MTX-injected mice. Results are expressed as mean ± SEM, (*n* = 6). a indicates significant (*p* < 0.05) vs. control, while b indicates significant (*p* < 0.05) vs. MTX.

**Figure 2:**
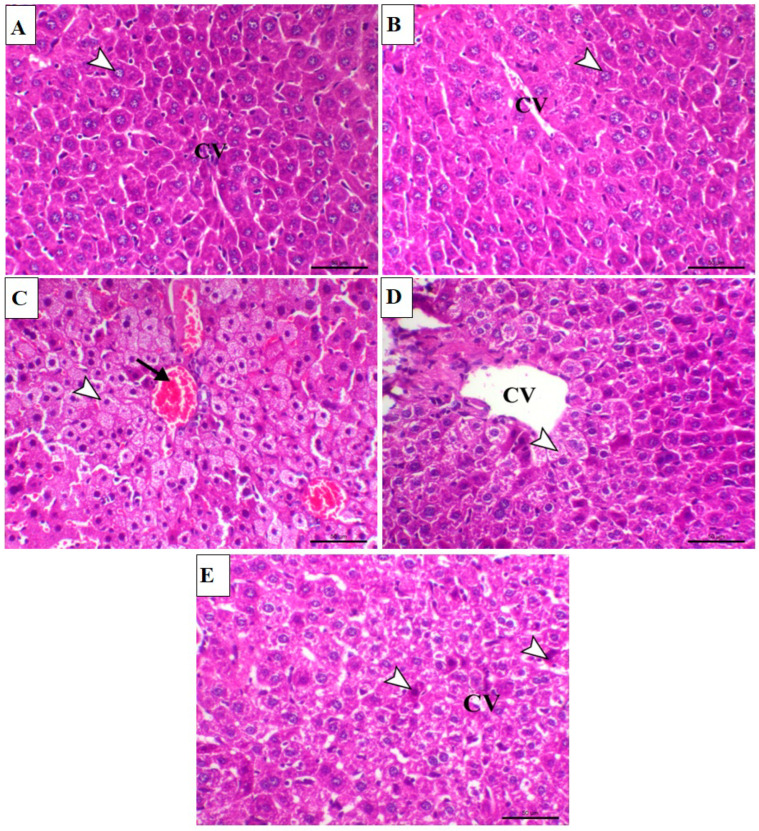
PU ameliorates MTX-induced histopathological alterations in the liver. Image of hepatic sections from (**A**) control mice showing normal hepatic cells (arrowhead) arranged in cords and separated with sinusoids around the central vein (CV); (**B**) PU-treated animals demonstrating normal hepatocytes (arrowhead) around the central vein (CV), (**C**) MTX-treated animals demonstrating congestion of hepatic blood vessels (arrow) and marked granular hepatic vacuolation associated with marked nuclear pyknosis (arrowhead); (**D**) MTX-administered mice pre-treated with 25 mg PU showing marked decreased hepatic vacuolation which restricted mostly centrilobular (arrowhead) (CV indicates central vein); and (**E**) MTX-administered animal group pre-treated with 50 mg PU demonstrating noticeable decrease in hepatic vacuolation with few cellular apoptosis (arrowheads) (CV indicates central vein) (H&E, X200, Scale bar = 50 µm).

To investigate the protective effect of PU on MTX-induced liver injury, we evaluated liver function markers levels (Figure 1A–D) and histological changes (Figure 2) in both PU-treated and untreated mice. MTX resulted in a significant (*p* < 0.05) increase in serum aspartate aminotransferase (AST) (Figure 1A), alanine transaminase (ALT) (Figure 1B), alkaline phosphatase (ALP) (Figure 1C), and lactate dehydrogenase (LDH) (Figure 1D) activities when compared to control mice. The treatment of MTX-intoxicated mice with PU significantly (*p* < 0.05) ameliorated serum AST, ALT, ALP, and LDH activities. PU alone had no effects on the liver enzymes in the healthy mice.

The effect of PU on MTX-induced liver injury was further evaluated by examining the hematoxylin and eosin (H&E)-stained liver sections of both PU-treated and untreated mice. As shown in Figure 2A,B, histological examination of liver sections from control and PU-treated mice demonstrated normal hepatic cells arranged in cords and separated with sinusoids around the central vein. Examination of sections from the liver of MTX-treated mice showed congestion of hepatic blood vessels and marked granular hepatic vacuolation associated with marked nuclear pyknosis (Figure 2C). These histopathological changes were remarkably attenuated when MTX-injected mice were treated with both doses of PU (Figure 2D,E).

### 2.2. PU Attenuates Oxidative Stress and Enhances Antioxidants Defenses in Liver of MTX-Treated Mice

**Figure 3:**
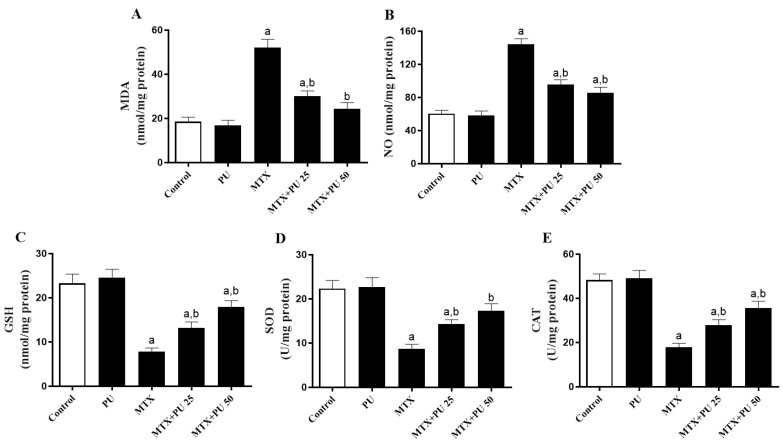
PU reduces hepatic levels of oxidative stress markers in mice exposed to MTX. Pre-treatment with PU decreased hepatic (**A**) MDA and (**B**) NO levels, elevated (**C**) GSH level, and (**D**) SOD and (**E**) CAT activities in MTX-injected mice. Results are expressed as mean ± SEM, (*n* = 6). a indicates significant (*p* < 0.05) vs. control, while b indicates significant (*p* < 0.05) vs. MTX.

The levels of hepatic malondialdehyde (MDA) and nitric oxide (NO) were significant (*p* < 0.05) in the mice intoxicated with MTX (Figure 3A,B, respectively); meanwhile, this group exhibited a significant decrease (*p* < 0.05) in the liver-reduced glutathione (GSH) contents (Figure 3C), as well as superoxide dismutase (SOD) and catalase (CAT) activities (Figure 3D,E, respectively). The PU treatment of MTX-injected mice significantly (*p* < 0.05) ameliorated MDA and NO levels and restored antioxidants in the MTX-induced liver. Normal mice that received PU alone had no effects on MDA and NO contents and antioxidants.

### 2.3. PU Suppresses the MTX-Induced Hepatic Inflammation in Mice

**Figure 4:**
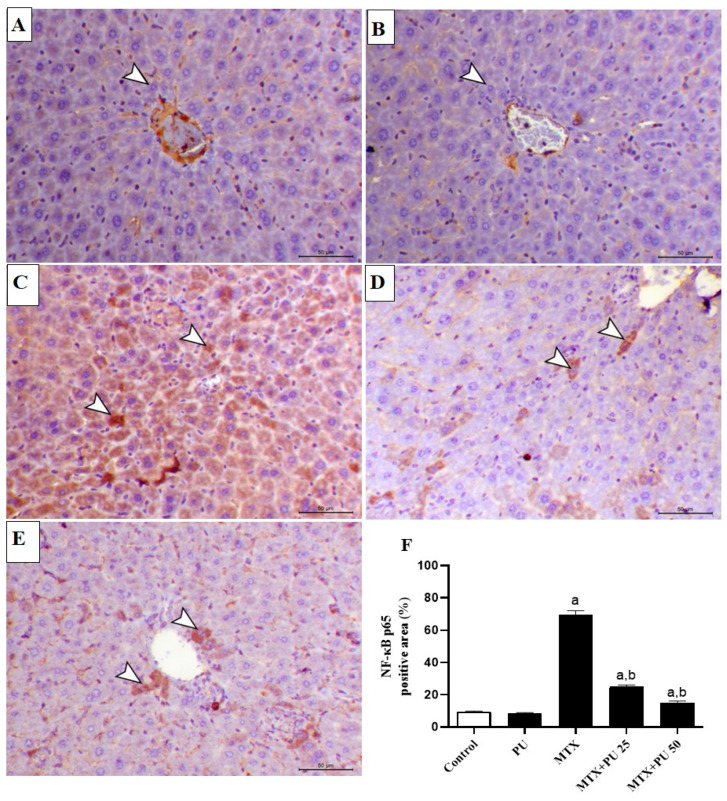
PU attenuates hepatic inflammatory reaction in MTX-administered mice. Photomicrographs of microscopic field from liver of (**A**) control and (**B**) PU-treated mice showing minimal immunoexpression of NF-κB p65 in the hepatic tissues (arrowheads); (**C**) MTX-injected mice demonstrating marked immunoexpression of cytoplasmic and nuclear hepatocytes’ NF-κB p65 (arrowheads); (**D**) MTX-injected mice pre-treated with 25 mg PU demonstrating decreased hepatocytes’ NF-κB p65 immunoexpression (arrowheads); and (**E**) MTX-injected animals pre-treated with 50 mg PU demonstrating noticeable decreased hepatocytes’ NF-κB p65 immunoexpression (arrowheads) (IHC, X200, Scale bar = 50 µm). (**F**) Image analysis of hepatocytes’ NF-κB p65 immunostaining demonstrating significant rise of NF-κB p65 in MTX-injected mice and significant decline of NF-κB p65 in mice pretreated with both doses of PU before MTX injection. Results are expressed as mean ± SEM, (*n* = 6). a indicates significant (*p* < 0.05) vs. control, while b indicates significant (*p* < 0.05) vs. MTX.

**Figure 5:**
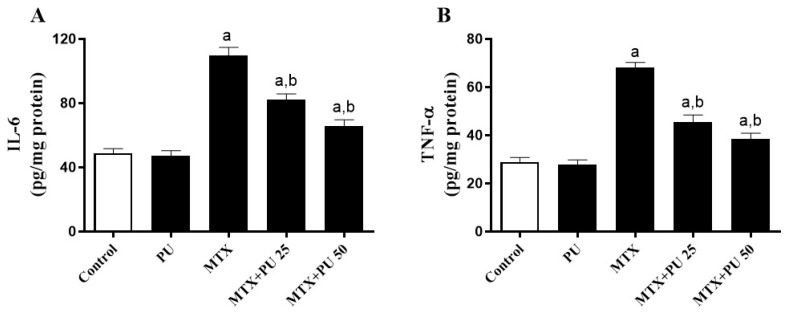
PU reduces hepatic (**A**) IL-6 and (**B**) TNF-α in MTX-injected mice. Results are expressed as mean ± SEM, (*n* = 6). a indicates significant (*p* < 0.05) vs. control, while b indicates significant (*p* < 0.05) vs. MTX.

Inflammatory response plays a crucial role in the pathogenicity of MTX-induced liver toxicity. Inflammation was significantly elevated in the hepatic tissue of MTX-intoxicated mice when compared to the control animal group. This was indicated by a considerable (*p* < 0.05) rise in the hepatic NF-κB p65 expression (Figure 4A–F) as well as pro-inflammatory cytokine levels, interleukin-6 (IL-6) (Figure 5A), and tumor necrosis factor-alpha (TNF-α) (Figure 5B). Treatment of MTX-injected mice with both doses of PU significantly (*p* < 0.05) ameliorated NF-κB p65 expression and IL-6 and TNF-α levels in the liver. However, PU alone did not affect the above-mentioned markers in healthy mice.

### 2.4. PU Mitigates the MTX-Induced Apoptosis in the Liver

**Figure 6:**
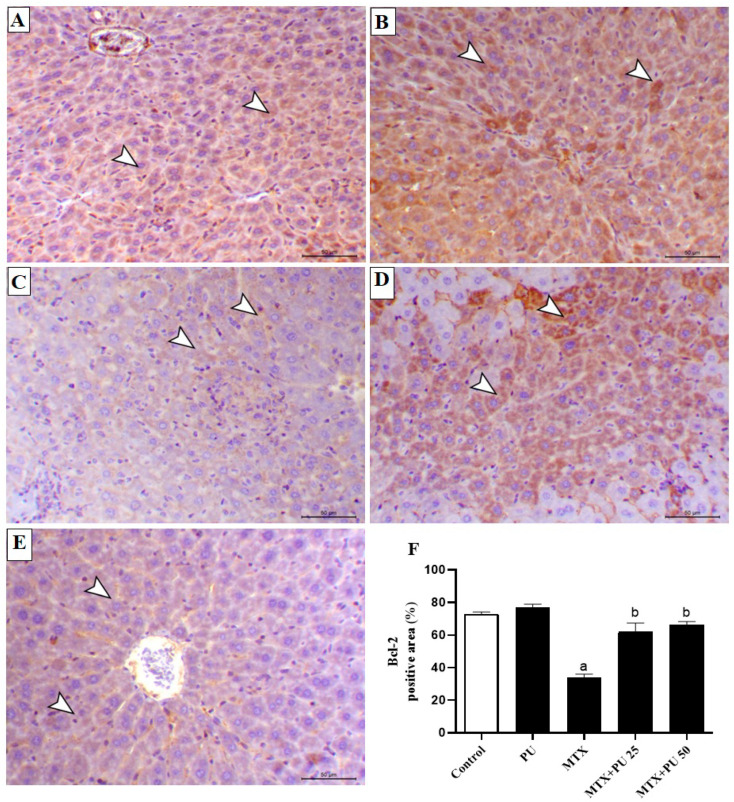
PU attenuates hepatic Bcl-2 in MTX-injected mice. Image of microscopic field of hepatic tissue from (**A**) control and (**B**) PU-treated mice showing high level of hepatocytes Bcl-2 immunoexpression (arrowheads), (**C**) MTX- injected mice showing marked decrease in hepatocytes’ Bcl-2 immunoexpression (arrowheads); (**D**) MTX-injected mice pre-treated with 25 mg PU demonstrating increased Bcl-2 immunoexpression within the hepatic tissues (arrowheads); and (**E**) MTX-injected mice pre-treated with 50 mg PU showing noticeable increase in hepatocytes’ Bcl-2 immunoexpression (arrowheads) (IHC, X200, Scale bar = 50 µm). (**F**) Image analysis of hepatic Bcl-2 immunostaining demonstrating significant decline of Bcl-2 level in MTX-injected mice and significant elevation of Bcl-2 level in both doses of PU-pretreated mice exposed to MTX. Results are expressed as mean ± SEM, (*n* = 6). a indicates significant (*p* < 0.05) vs. control, while b indicates significant (*p* < 0.05) vs. MTX.

**Figure 7:**
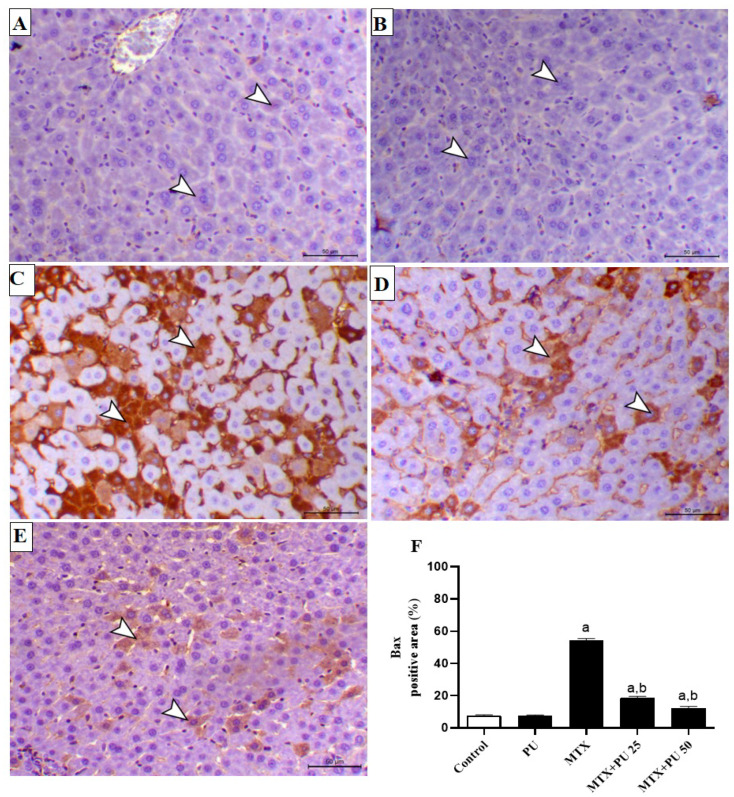
PU decreases hepatic Bax in MTX-administered mice. Image of hepatic microscopic field of (**A**) control and (**B**) PU-treated mice showing scanty hepatocytes’ expression of Bax (arrowheads); (**C**) MTX-injected mice showing marked immunoexpression of Bax within the hepatic tissues (arrowheads); (**D**) MTX-injected mice pre-treated with 25 mg PU demonstrating decreased Bax immunoexpression within the hepatic tissues (arrowheads); and (**E**) MTX-injected mice pre-treated with 50 mg PU demonstrating noticeable decline in hepatocytes’ Bax immunoexpression (arrowheads) (IHC, X200, Scale bar = 50 µm). (**F**) Image analysis of hepatic Bax immunostaining demonstrating significant elevation of Bax level in MTX-injected mice and significant decline of Bax level in MTX-exposed mice pretreated with both doses of PU. Results are expressed as mean ± SEM, (*n* = 6). a indicates significant (*p* < 0.05) vs. control, while b indicates significant (*p* < 0.05) vs. MTX.

**Figure 8:**
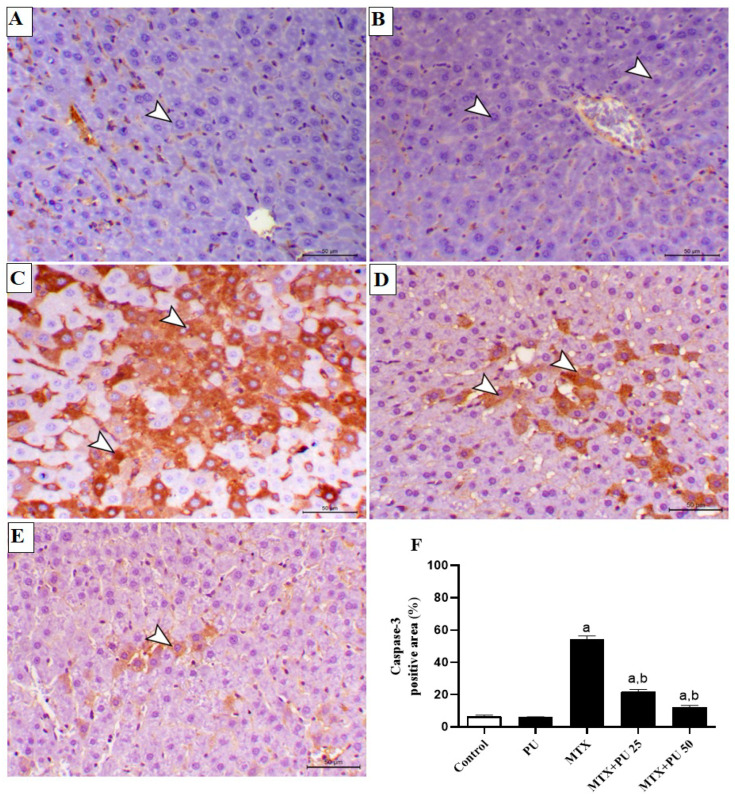
PU decreases hepatic caspase-3 in MTX-injected mice. Images of hepatic microscopic fields from (**A**) control and (**B**) PU-treated animals demonstrating slight immunoexpression of caspase-3 within the hepatic tissues (arrowheads); (**C**) MTX-administered mice showing marked immunoexpression of caspase-3 within the hepatic tissues (arrowheads); (**D**) MTX-intoxicated mice pre-treated with 25 mg PU demonstrating decreased caspase-3 immunoexpression within the hepatic tissues (arrowheads); and (**E**) MTX-injected mice pre-treated with 50 mg PU showing noticeable decreased hepatocytes’ caspase-3 immunoexpression (arrowheads) (IHC, X200, Scale bar = 50 µm). (**F**) Image analysis of hepatocytes’ caspase-3 immunostaining of mice demonstrating significant elevation in MTX-injected mice and significant decline in mice treated with both doses of PU. Results are expressed as mean ± SEM, (*n* = 6). a indicates significant (*p* < 0.05) vs. control, while b indicates significant (*p* < 0.05) vs. MTX.

To further assess the preventive impact of PU on MTX-injured liver, we determined the expression levels of Bax, Bcl-2, and caspase-3 in hepatic tissue by immunohistochemistry (IHC) staining. There was a significant (*p* < 0.05) decline in the level of Bcl-2 expression (Figure 6A–F), with concordant marked (*p* < 0.05) elevation of the expression levels of Bax (Figure 7A–F) and caspase-3 (Figure 8A–F) in liver of MTX-injected mice. This imbalance in the hepatic contents of Bax, Bcl-2, and caspase-3 were remarkably (*p* < 0.05) ameliorated when mice were pretreated with both doses of PU before MTX exposure. PU alone did not affect the expression levels of the above-mentioned apoptosis regulatory proteins in the liver.

### 2.5. PU Upregulates Hepatic Nrf2/Heme Oxygenase 1 (HO-1) in MTX-Treated Mice

**Figure 9:**
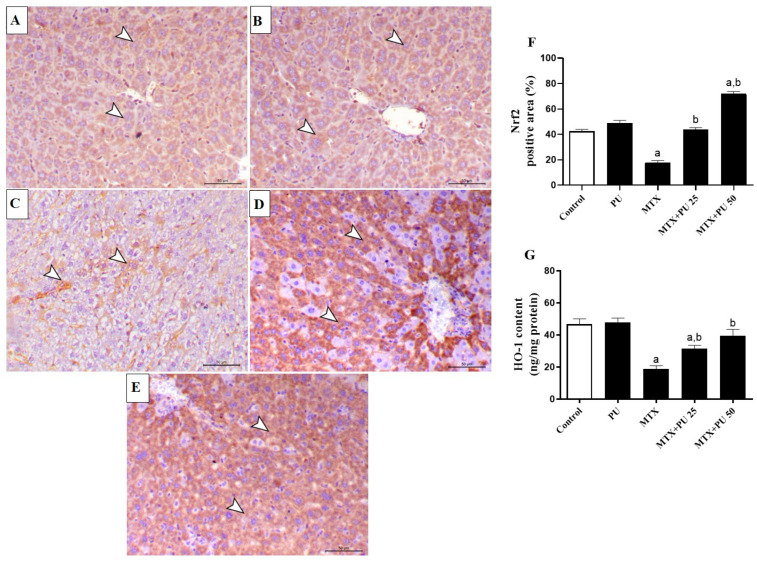
PU upregulates hepatic Nrf2/HO-1 signaling in MTX-injected animals. Images of hepatic microscopic fields from (**A**) control and (**B**) PU-treated mice showing noticeable immunoexpression of hepatocytes’ Nrf2 (arrowheads); (**C**) MTX-injected mice demonstrating considerable decrease in hepatocytes’ Nrf2 immunoexpression (arrowheads); (**D**) MTX-injected mice pre-treated with 25 mg PU demonstrating an elevation of Nrf2 immunoexpression within the hepatic tissues (arrowheads); and (**E**) MTX-injected animals pre-treated with 50 mg PU showing marked increase in Nrf2 immunoexpression within the hepatic tissues (arrowheads) (IHC, X200, Scale bar = 50 µm). (**F**) Image analysis of hepatic Nrf2 immunostaining showing remarkable upregulation in MTX-injected mice treated with both doses of PU. (**G**) PU markedly attenuates hepatic levels of HO-1 in MTX-injected mice. Results are expressed as mean ± SEM, (*n* = 6). a indicates significant (*p* < 0.05) vs. control, while b indicates significant (*p* < 0.05) vs. MTX.

Since targeting Nrf2 is suggested to attenuate oxidative damage and inflammation, changes in its expression in the liver of both PU-treated and untreated mice were determined by IHC staining (Figure 9A–F). MTX-treated mice demonstrated significantly (*p* < 0.05) downregulated hepatic expressions of Nrf2 as compared to the control animal group. Such downregulation of the hepatic Nrf2 expression was significantly (*p* < 0.05) attenuated by PU pre-treatment (Figure 9A–F) of MTX-injected mice. In addition, both doses of PU significantly (*p* < 0.05) attenuated HO-1 in the mouse liver (Figure 9G). Normal mice that received PU alone had no effects on Nrf2 expression and HO-1 content in the liver.

## 3. Discussion

The antifolate metabolite MTX is one of the most effective and widely used drugs in the management of a range of malignancies and autoimmune disorders [4,51]. However, it can induce multi-organ toxicity, including hepatotoxicity, which involves increasing ROS generation and activating pro-inflammatory and cell death pathways, limiting its clinical use [8,9,19]. Therefore, the development of efficient promising protective approaches to prevent MTX hepatotoxicity is needed. In this study, we demonstrated that PU mitigated MTX-induced liver injury via attenuating oxidative tissue injury, inflammatory response, and cell death, and upregulating Nrf2 in the liver tissue of mice.

Consistent with several studies [19,52,53,54], MTX-induced liver damage was demonstrated in this study by increased serum levels of transaminases (ALT and AST), ALP, and LDH, as well as various histological abnormalities in liver tissues. Clinically, elevated blood liver enzymes are frequently linked with MTX therapy, indicating hepatocellular degeneration and necrosis [15]. MTX administration has been associated with acute hepatocellular necrosis, steatosis, cholestasis, fibrosis, and cirrhosis [2,52]. The localization of histopathological changes in the pericentral hepatocytes may suggest that MTX is more abundant in this region than in the periportal and midzonal lobular zones. This could be attributable to increased glutamate uptake by pericentral hepatocytes, which enhances MTX polyglutamate production by folylpolyglutamyl synthatase and boosts intracellular MTX accumulation [55]. Furthermore, since the pericentral area is the furthest zone from the arterial blood supply, it has relatively lower levels of oxygen and GSH, making it more vulnerable to hypoxia and oxidative damage [56,57]. PU pre-treatment, on the other hand, demonstrated a potent hepatoprotective effect against MTX-induced hepatotoxicity, as shown by reducing both serum markers and histopathological features of liver injury. These findings are consistent with numerous previous reports that showed the hepatoprotective potential of PU against liver injury induced by drugs and chemicals like cyclophosphamide [39], acetaminophen [37], and tetrachloromethane [41].

Increased oxidative damage of the liver has been described as an important primary mechanism leading to the development of MTX-induced hepatotoxicity [21,22,27]. In hepatocytes, the enzyme folylpolyglutamyl synthetase converts MTX to MTX polyglutamates by adding up to six glutamate residues to MTX and increasing its intracellular retention, which triggers ROS overproduction and oxidative damage in liver tissue [13,15]. In turn, gamma-glutamyl hydrolase converts MTX polyglutamates back to MTX by removing glutamates from the polyglutamates, which are subsequently eliminated from cells by ATP-binding cassette transporters [58]. Furthermore, at a high dose of MTX, the amount of MTX is converted to the toxic metabolite 7-hydroxymethotrexate by the action of aldehyde oxidase as a dose-dependent alternate pathway [16,17,50]. Oxidative stress can cause potentially harmful events in the cell, including LPO, protein oxidation, and oxidative DNA damage, which are considered crucial factors in triggering the pathologic changes associated with MTX hepatotoxicity [5,19,26,53,59]. Herein, the liver of MTX-treated mice showed increased MDA and NO contents, along with a marked decline in GSH content and SOD and CAT activities. LPO is considered a destructive process that affects cellular membranes and causes membrane permeability and fluidity changes with significant biological consequences [60]. Furthermore, protein oxidation causes protein unfolding, aggregation, or fragmentation, as well as enzyme and other protein inactivation, all of which result in protein degradation and, ultimately, cell death [61]. Importantly, peroxynitrite, a powerful oxidant formed by the interaction of superoxide anion with NO, aggravates oxidative damage by further oxidation of cellular components such as lipids, DNA, and proteins, resulting in cell death [62]. Therefore, attenuating oxidative stress and enhancing antioxidant defenses might represent powerful therapeutic tools for the prevention of hepatotoxicity induced by MTX. In the present study, PU treatment of MTX-injected mice markedly attenuated MDA and NO contents and boosted antioxidants in the liver. Consistent with our findings, PU decreased MDA and restored GSH contents and CAT and SOD activities in the kidney of a rat model of cisplatin-induced induced AKI [34]. Another study showed that PU treatment reduced the MDA level and increased SOD and glutathione peroxidase (GPx) activities in the liver of CCl_4_-induced hepatic injury [41]. PU was also able to prevent cyclophosphamide-induced oxidative tissue injury in the liver by decreasing MDA and NO levels and increasing total antioxidant capacity (TAC) in rats [63].

Multiple lines of evidence indicate that the MTX-induced ROS production may trigger an inflammatory response through activating the redox-sensitive factor NF-κB that induces the release of pro-inflammatory mediators resulting in increased inflammation and associated oxidative stress, hence promoting the progression of liver injury and dysfunction [15,53,54]. Consistent with several studies [15,21,22], the liver of MTX-injected mice showed increased NF-κB p65 expression and TNF-α and IL-6 levels in the liver. Indeed, increased oxidative stress and inflammation may promote the activation of stress signaling pathways facilitating apoptotic cell death in the liver [64,65]. In the present study, the MTX-treated mice showed increased expression of Bax and caspase-3 and decreased expression of Bcl-2 in the liver. These findings were supported by previous studies where MTX injection was associated with increased apoptosis in the liver [21,22,53]. The most likely trigger of MTX-induced apoptosis is sustained ROS production after MTX exposure that culminates in the dissipation of mitochondrial membrane potential and cytochrome *c* release which ultimately induces the execution phase of caspase-3-dependent apoptosis [15,25,27]. Thus, prevention of the MTX-induced ROS overproduction and NF-κB activation can attenuate apoptosis and consequently protect against liver injury and dysfunction induced by MTX. PU suppressed NF-κB and decreased TNF-α, and IL-6 in the liver of MTX-injected mice, demonstrating its anti-inflammatory activity. Furthermore, PU prevented MTX-induced apoptosis in the liver as shown by the decreased Bax and caspase-3 and increased Bcl-2 expressions. Consistently, PU protected against inflammation and apoptosis where it suppressed NF-κB, TNF-α, IL-1β, Bax, and caspase-3 and enhanced Bcl-2 in the kidney of cisplatin-induced nephrotoxicity [34]. Additionally, PU prevented cyclophosphamide inflammation and apoptosis in the liver through attenuating NF-κB p65, TNF-α, IL-1β, Bax/Bcl-2 ratio, inducible nitric oxide synthase, and caspases 3 and 9 levels [39]. Furthermore, PU decreased ROS formation and inhibited apoptosis in palmitate-mediated lipotoxicity in HepG2 cells through modulating cytochrome *c* release, Bax mitochondrial translocation, and caspase-3 activation [66]. Taken together, the suppressive effect of PU on MTX-induced apoptosis in the liver appears to be attributed, at least in part, to its potential inhibitory effects on ROS overproduction and inflammatory response.

It has extensively been suggested that the activation of Nrf2 is crucial in protecting against the development of drug-induced hepatotoxicity through the neutralization of ROS in the cell and the upregulation of antioxidant and cytoprotective genes [53,67,68]. It has been reported that Nrf2 activation prevented drug-induced liver injury through attenuating oxidative tissue damage and inflammatory response [67,69]. In contrast to these studies, Lv et al. [70], using Nrf2-deficient mice, indicated that the inhibition of Nrf2 may be deleterious and increase susceptibility to acetaminophen-induced hepatotoxicity. Therefore, interventions aiming at augmenting Nrf2 signaling can be of significant therapeutic benefit against MTX-induced hepatotoxicity. Accumulating evidence indicates that the use of Nrf2-activating natural compounds has shown effective therapeutic effects against MTX-induced liver injury without interfering with its anticancer effectiveness [53,54]. Herein, PU treatment effectively upregulated Nrf2 in the liver of MTX-injected mice. Accordingly, a recent study showed that PU treatment prevented cisplatin-induced oxidative tissue injury and inflammatory response in the kidney possibly via activating Nrf2-signaling pathway [34]. Similar findings also indicated that PU increased Nrf2 and HO-1 expression to prevent lipopolysaccharides (LPS)-induced oxidative stress in macrophages by reducing ROS and NO generation and increasing SOD1 expression [71]. Additionally, PU effectively ameliorated free fatty acids (FFA)-induced lipotoxicity in HepG2 cells by activating the Nrf2-signaling pathway [66].

The findings of this study showed the hepatoprotective effects of PU against MTX-induced liver injury; however, it has some limitations. Although PU attenuated MDA and NO levels and boosted antioxidant defenses in the liver, we didn’t measure its effect on ROS generation. This study showed that PU upregulated Nrf2 and HO-1 in the liver of MTX-intoxicated mice; however, it was unable to evaluate the effect of hepatic expression of other Nrf2-related antioxidant enzymes. While this study clearly indicated the downregulation and upregulation of some regulatory proteins as depicted by IHC and ELISA, we did not validate it by RT-qPCR to demonstrate the relationship between protein and mRNA levels.

## 4. Materials and Methods

### 4.1. Animals

Thirty Swiss albino mice weighing 23–25 g were used in this study. All animals were housed under standard conditions (temperature, 23 ± 2 °C; and relative humidity, 50 ± 10%) with a 12 h light/dark cycle. They were allowed to acclimatize for a week before beginning the experiment, and they were fed a standard chow diet with unrestricted access to water. Animal handling and related protocols were validated by the panel of animal research ethics at Al-Hussein Bin Talal University (AHU-198/2019) and were conducted in compliance with National Institutes of Health legislation (NIH publication No. 85–23, revised 2011).

Physiological saline and 0.5% carboxymethyl cellulose (CMC) were used as vehicle solutions to dissolve the MTX (Shanxi PUDE, Datong, China) and the PU (Santa Cruz Biotechnology, Dallas, TX, USA), respectively [34].

### 4.2. Experimental Design

The included animals were divided into five groups (*n* = 6). The control mice were administered with 0.5% CMC for 10 days and injected intraperitoneally (i.p.) once with physiological saline on day 7. The mice in the second group (PU) were administered with PU (50 mg/kg) orally for 10 days and one injection (i.p.) of physiological saline on day 7. Meanwhile, groups III (MTX), IV (PU 25 mg/kg + MTX), and V (PU 50 mg/kg + MTX) animals were administered orally with 0.5% CMC, 25 mg/kg PU, or 50 mg/kg PU, respectively, for 10 days and a single dose of MTX (20 mg/kg, i.p.) at day 7. The PU and MTX doses were determined based on the previous reports of Aladaileh et al. [34] and Mahmoud et al. [53], respectively.

The animals were anesthetized on the 11th day of the experiment using a ketamine–xylazine combination (100 mg/kg, 10 mg/kg, respectively, i.p.). A cardiac puncture was performed for blood sampling, then serum was separated for biochemical assessment. Instantly, the mice were dissected, and the liver tissues were isolated and washed in 50 mM cold phosphate buffer (PBS) (pH 7.0). Parts of liver specimens were fixed in 10% neutral buffered formalin for histological examination. The rest of the liver tissues were homogenized in cold PBS (10% *w*/*v*), centrifuged, and the clear homogenate was collected and stored at −20 °C for further analysis of biochemical parameters.

### 4.3. Estimation of Markers of Liver Function

Activities of liver function enzymes including AST, ALT, ALP, and LDH were determined in the serum of both PU-treated and untreated mice using kits procured from Spinreact (Girona, Spain).

### 4.4. Assessment of Oxidative Stress Markers and Antioxidant Contents in Liver Tissues

The hepatic levels of oxidative stress markers including MDA and NO were determined according to the methods described by Ohkawa et al. [72] and Green et al. [73], respectively. In addition, the activities of the antioxidant contents including SOD [74] and CAT [75], as well as the levels of GSH [76] were estimated in the liver of both PU-treated and untreated mice. A specific ELISA kit (MyBioSource, San Diego, CA, USA) was used for the measurement of hepatic HO-1 content according to the protocol provided.

### 4.5. Estimation of Pro-Inflammatory Cytokines in Liver

Following the manufacturer’s instructions of R&D Systems (Minneapolis, MN, USA) ELISA kits, the levels of pro-inflammatory cytokines, including TNF-α and IL-6, were estimated in hepatic tissues of both PU-treated and untreated mice.

### 4.6. Histological Examination of Liver Sections

The formalin-fixed specimens were dehydrated, cleared in xylene, and embedded in paraffin. Next, 5-μm slices were prepared using a rotary microtome before the sections were deparaffinization and rehydration. Then, they were subjected to H&E for routine histopathological examination and the histopathological changes were observed using light microscopy and evaluated in a blinded manner by a histopathologist.

### 4.7. Immunohistochemistry

For IHC, the deparaffinized and hydrated sections were treated with 0.05 M citrate buffer (pH 6.8) for antigen retrieval followed by 0.3% hydrogen peroxide. The nonspecific antigen-antibody binding was blocked through the addition of normal serum for 20 min. The sections were washed in PBS and probed overnight at 4 °C with anti-NF-κB p65 (ThermoFisher, Waltham, MA, USA), anti-Bax (Abcam, Cambridge, MA, USA), anti-Bcl-2 (Abcam, Cambridge, MA, USA), anti-caspase-3 (ThermoFisher, Waltham, MA, USA), and anti-Nrf2 (ThermoFisher, Waltham, MA, USA). After washing in PBS, anti-mouse secondary antibodies were added to the slides, and DAB was used for color development. Then, the sections were counterstained with Mayer’s hematoxylin, and examined under a light microscope. The staining labelling indices of the caspase-3 and NF-κB p65 were presented as a percentage equivalent field of positive control expression. The immunostaining intensity of anti-Bcl-2 and anti-Nrf2 antibodies was determined through a percent of the positive area using image J analysis software (NIH, Bethesda, MD, USA).

### 4.8. Analysis of Data

The mean and S.E.M are used to express the results of this study. GraphPad Prism 7 software (San Diego, CA, USA) was used to determine all statistical differences among groups using analysis of variance (ANOVA) followed by Tukey’s post hoc test. A *p* value of less than 0.05 was deemed significant.

## 5. Conclusions

The observations of this study introduced evidence that PU can be of significant prophylactic benefit against MTX hepatotoxicity by decreasing oxidative tissue injury, inflammation, and cell death in the liver. These prophylactic effects were associated with upregulating Nrf2 and boosting antioxidant defenses. Therefore, PU could be suggested as the potential for a new preventive approach targeting MTX hepatotoxicity and perhaps other toxic effects, pending further studies exploring its exact protective mechanism(s) to be conducted.

## Data Availability

Data analyzed or generated during this study are included in this manuscript.
